# Biophysical basis of the sound analog membrane potential that underlies coincidence detection in the barn owl

**DOI:** 10.3389/fncom.2013.00102

**Published:** 2013-11-08

**Authors:** Go Ashida, Kazuo Funabiki, Catherine E. Carr

**Affiliations:** ^1^Department of Biology, University of Maryland, College ParkMD, USA; ^2^Systems Biology, Osaka Bioscience InstituteSuita, Japan; ^3^Division of Biology, California Institute of TechnologyPasadena, CA, USA

**Keywords:** phase-locking, sound localization, auditory brainstem, periodic signals, oscillation, owl

## Abstract

Interaural time difference (ITD), or the difference in timing of a sound wave arriving at the two ears, is a fundamental cue for sound localization. A wide variety of animals have specialized neural circuits dedicated to the computation of ITDs. In the avian auditory brainstem, ITDs are encoded as the spike rates in the coincidence detector neurons of the nucleus laminaris (NL). NL neurons compare the binaural phase-locked inputs from the axons of ipsi- and contralateral nucleus magnocellularis (NM) neurons. Intracellular recordings from the barn owl's NL *in vivo* showed that tonal stimuli induce oscillations in the membrane potential. Since this oscillatory potential resembled the stimulus sound waveform, it was named the sound analog potential (Funabiki et al., 2011). Previous modeling studies suggested that a convergence of phase-locked spikes from NM leads to an oscillatory membrane potential in NL, but how presynaptic, synaptic, and postsynaptic factors affect the formation of the sound analog potential remains to be investigated. In the accompanying paper, we derive analytical relations between these parameters and the signal and noise components of the oscillation. In this paper, we focus on the effects of the number of presynaptic NM fibers, the mean firing rate of these fibers, their average degree of phase-locking, and the synaptic time scale. Theoretical analyses and numerical simulations show that, provided the total synaptic input is kept constant, changes in the number and spike rate of NM fibers alter the ITD-independent noise whereas the degree of phase-locking is linearly converted to the ITD-dependent signal component of the sound analog potential. The synaptic time constant affects the signal more prominently than the noise, making faster synaptic input more suitable for effective ITD computation.

## Introduction

The ability to tell the direction of the sound source, or sound localization, is a fundamental auditory function in many animal species. Among various species examined (see Klump, 2000; Heffner and Heffner, 2003; for reviews), the barn owl, which can locate its prey in the total darkness purely on the basis of acoustic cues (Payne, 1971; Konishi, 1973), shows great sound localization acuity, with a minimum discriminable angle of a few degrees (Knudsen et al., 1979; Bala et al., 2003). The auditory system of the barn owl computes the interaural time difference (ITD) to determine the azimuthal location of the sound source (Konishi, 1993). In birds, the ITD, or the time difference of sound arrival between two ears, is computed in a specialized neural circuit: axons from the cochlear nucleus magnocellularis (NM) form delay lines, and neurons of the nucleus laminaris (NL) detects coincident inputs from ipsi- and contralateral NM axons (Jeffress, 1948; Carr and Konishi, 1990). Physiological studies showed that barn owls' NL neurons vary their discharge rates with changes in ITDs of less than 10 μs (Carr and Konishi, 1990; Peña et al., 1996). A wide variety of highly-specialized cellular, synaptic, and network mechanisms underlie this temporal acuity (see Grothe et al., 2010; Ashida and Carr, 2011, for recent reviews).

**Figure 1:**
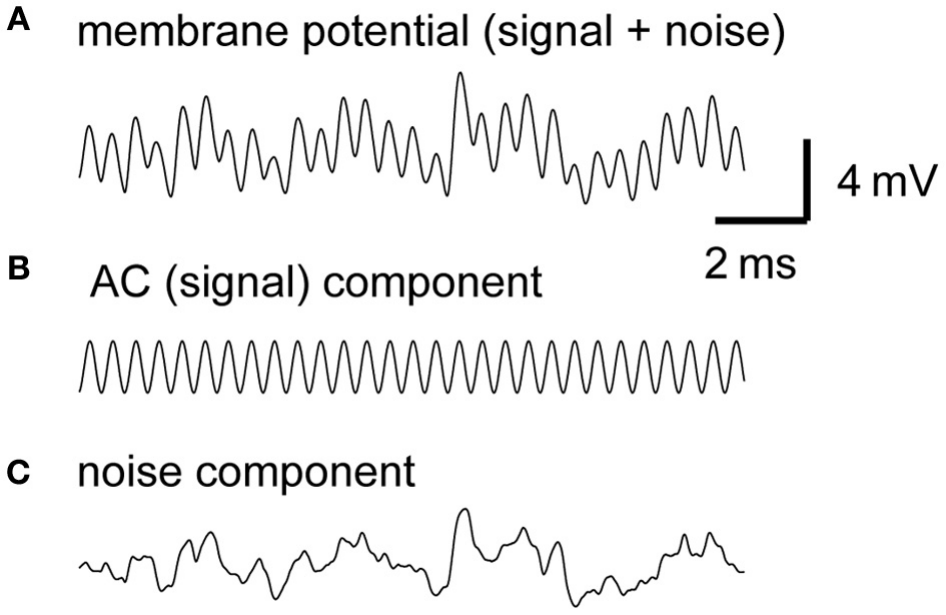
**Example traces of the model membrane potential with default parameters**. The default parameters used for this example are summarized in Table 1. **(A)** Simulated membrane potential oscillating at 4 kHz. **(B)** AC component of the simulated membrane potential. Amplitude = 1.25 mV (see Materials and Methods for definition). Average peak-to-peak height = 2.50 mV. **(C)** Noise component of the simulated membrane potential. Amplitude (measured by the time-averaged standard deviation) is 1.03 mV in this example.

In our previous reports, we investigated the cellular properties of owl NL neurons using *in vivo* intracellular recordings (Funabiki et al., 2011) and modeling (Ashida et al., 2007; Funabiki et al., 2011). When tonal stimuli were presented, the membrane potential of the NL neuron oscillated at the same frequency as the stimulus tone and was thus named the “sound analog potential” (Funabiki et al., 2011). The amplitude of this sound-induced oscillation changes periodically with ITD, showing an almost linear relationship with the spike rate of the NL neuron. Previous modeling results demonstrated that convergence of phase-locked excitatory synaptic inputs from NM fibers gives rise to oscillatory membrane potentials (Kempter et al., 1998; Slee et al., 2010; see Figure 1A for an example) and that the sound analog potential observed in NL can be quantitatively reproduced if biologically plausible parameters are chosen (Ashida et al., 2007; Funabiki et al., 2011). How presynaptic, synaptic, and postsynaptic properties, however, affect the formation of the sound analog potential remains to be elucidated.

In the accompanying paper, we focused on the theoretical relationship between phase-locked inputs and membrane potential oscillations (Ashida et al., 2013). The key factors which can affect the formation of the sound analog potentials include (1) the frequency of the tonal stimulus, (2) the mean firing rate of NM neurons, (3) the number of converging NM fibers per NL neuron, (4) the average degree of phase-locking of these fibers, (5) the time scale of unitary synaptic input, and (6) the membrane properties of the NL neuron. Among these factors, the membrane properties have been extensively studied. A number of studies have shown that ITD coding in the avian NL and in the mammalian medial superior olive (MSO) is dynamically controlled by the low threshold potassium (K_LVA_) channels (Manis and Marx, 1991; Reyes et al., 1994; Svirskis et al., 2002; Rothman and Manis, 2003; Day et al., 2008; Gai et al., 2009; Jercog et al., 2010; Mathews et al., 2010), hyperpolarization-activated cation channels (Yamada et al., 2005; Khurana et al., 2011), and by optimized fast sodium channels (Kuba et al., 2006; Ashida et al., 2007; Scott et al., 2010). The accompanying paper (Ashida et al., 2013) examines the first key factor, the effect of tonal frequency on the formation of the sound analog potential. In the present paper, we focus on the remaining four factors (2–5 above) and systematically examine how the sound analog potential and ITD coding of the NL neuron depends on these presynaptic and synaptic factors.

## Results

### Phase-locked inputs and oscillatory potentials

Our model consists of NM fibers and a single-compartment NL cell body. The phase-locked spiking activity of each NM fiber is modeled as an inhomogeneous Poisson process with a periodic intensity function oscillating at the stimulus sound frequency. Spikes of converging NM fibers are summed and then modified by the synaptic and membrane filters, producing an oscillation in the membrane potential of the NL neuron, namely, the sound analog potential (Figure 1A). The mean synaptic input is referred to as the “DC” component, while the main oscillation component at the stimulus frequency is called the “AC” or “signal” component (Figure 1B) because the NL neuron changes its spike rate almost linearly with the amplitude of the AC component (Funabiki et al., 2011). All the other frequency components, including higher harmonics, are regarded as “noise” (Figure 1C) because they do not encode ITDs (Ashida et al., 2007; Slee et al., 2010). Analytical expressions that relate model parameters to the DC, AC, and noise components are summarized in Table 1. The accompanying paper (Ashida et al., 2013) shows that with default parameters (Table 1) the linear approximation gives good predictions for the DC, AC, and noise of simulated synaptic inputs and oscillatory membrane potentials. In the following sections, we examine how sound analog potentials are controlled by the number of presynaptic NM fibers, the mean firing rate of these fibers, their average degree of phase-locking measured by vector strength (*VS*), and the synaptic time scale measured by the half peak width of the unitary synaptic input. Note that, in our simulations, we assumed that ipsi- and contralateral NM inputs arrived perfectly in-phase, resulting in the maximum oscillation amplitude. The ITD-dependence of the sound analog potential will be examined in the section titled Implications for ITD coding.

**Table 1:** **Equations and parameter values for the model synaptic input**.

**Variable/parameter**	**Equation/value**
Modified Bessel function of order *n*	$I_{n}(\kappa )=\frac{1}{2\pi }\int_{-\pi }^{\pi }\text{exp}\left(\kappa \cos(x)\right)\cos(nx)dx$
von-Mises distribution function with concentration parameter κ	$p_{\kappa }(t)=\frac{1}{2\pi I_{0}(\kappa )}\text{ exp}\left(\kappa \cos(t)\right)=\frac{1}{2\pi }+\frac{1}{\pi }\sum_{n\;=\;1}^{\infty }\frac{I_{n}(\kappa )}{I_{0}(\kappa )}\cos(nt)$
Periodic intensity function for the inhomogeneous Poisson NM inputs	λ(*t*) = 2πλ_0_ *p*_κ_(2π*f*_*s*_*t*)
Timing of the *i*-th spike of the *m*-th NM fiber	*t*_*m*_^*i*^
Unitary synaptic input conductance (alpha function) with time constant τ	α (*t*) = (*Ht*/τ) exp (1 − *t*/τ) (*t* ≥ 0)
Area between α(*t*) and the *t*-axis	*S* = *eH*τ
Fourier transform of α(*t*)	$\left|F_{\alpha }(f)\right|=\left|\int_{0}^{\infty }\alpha (t)\text{ exp}(-2\pi ift)\;dt\right|=\frac{S}{1+{\left(2\pi f\tau \right)}^{2}}$
Total synaptic input conductance	$g_{\text{syn}}(t)=\sum_{m\;=\;1}^{M}\sum_{i\;=\;1}^{I_{m}}\alpha \left(t-t_{m}{}^{i}\right)$
Linear membrane impedance	|*Z*(*f*)|
DC component of the input conductance **(Equation 1)**	*D*_*G*_ = *SM*λ_0_
AC component of the input conductance **(Equation 2)**	$A_{G}=\frac{2rD_{G}}{1+{\left(2\pi f_{s}\tau \right)}^{2}}$
Noise component of the input conductance **(Equation 3)**	$N_{G}=\sqrt{M\lambda_{0}\int_{-\infty }^{\infty }{\left|F_{\alpha }(f)\right|}^{2}df}=\frac{D_{G}}{2\sqrt{M\lambda_{0}\tau }}$
AC component of the membrane potential **(Equation 4)**	$A_{V}=\frac{2rD_{G}}{1+{\left(2\pi f_{s}\tau \right)}^{2}}\left|E_{\text{syn}}-V_{0}\right|\left|Z\left(f_{s}\right)\right|$
Noise component of the membrane potential **(Equation 5)**	$N_{V}=\frac{D_{G}\left|E_{\text{syn}}-V_{0}\right|}{\sqrt{M\lambda_{0}}}\sqrt{\int_{-\infty }^{\infty }\frac{{\left|Z(f)\right|}^{2}}{{\left(1+{\left(2\pi f\tau \right)}^{2}\right)}^{2}}df}$
Equation for average potential *V*_0_	*g*_*L*_(*E*_*L*_ − *V*_0_) + *ḡ*_*K*_ *d*_∞_(*V*_0_)(*E*_*K*_ − *V*_0_) + *D*_*G*_(*E*_syn_ − *V*_0_) = 0
Stimulus sound frequency	*f*_*s*_ (default: *f*_*s*_ = 4000 Hz)
Mean spike rate of each NM fiber	λ_0_ (default: λ_0_ = 500 Hz)
Number of NM fibers converging onto one NL cell	*M* (default: *M* = 300 fibers)
Vector strength of phase-locked NM spikes	*r* = *I*_1_(κ)/*I*_0_(κ) (default: *r* = 0.6, κ = 1.516)
Half-peak-width of unitary input	*W* = 2.446τ (default: *W* = 0.1 ms; τ = 0.0409 ms)
Magnitude of unitary input	*H* = α (τ) (default: *H* = 1.3 nS)

The model equations and parameters are the same as those used in our previous (Funabiki et al., 2011) and accompanying (Ashida et al., 2013) papers. The number (M) and the mean spike rate (λ_0_) of the NM fiber are taken from previous anatomical (Carr and Boudreau, 1993) and physiological (Peña et al., 1996) studies. Equations 1–5, obtained in the accompanying paper (Ashida et al., 2013), describe how each model parameter affects the formation of the sound analog synaptic input and membrane potential.

### Mean spike rate of NM

**Figure 2:**
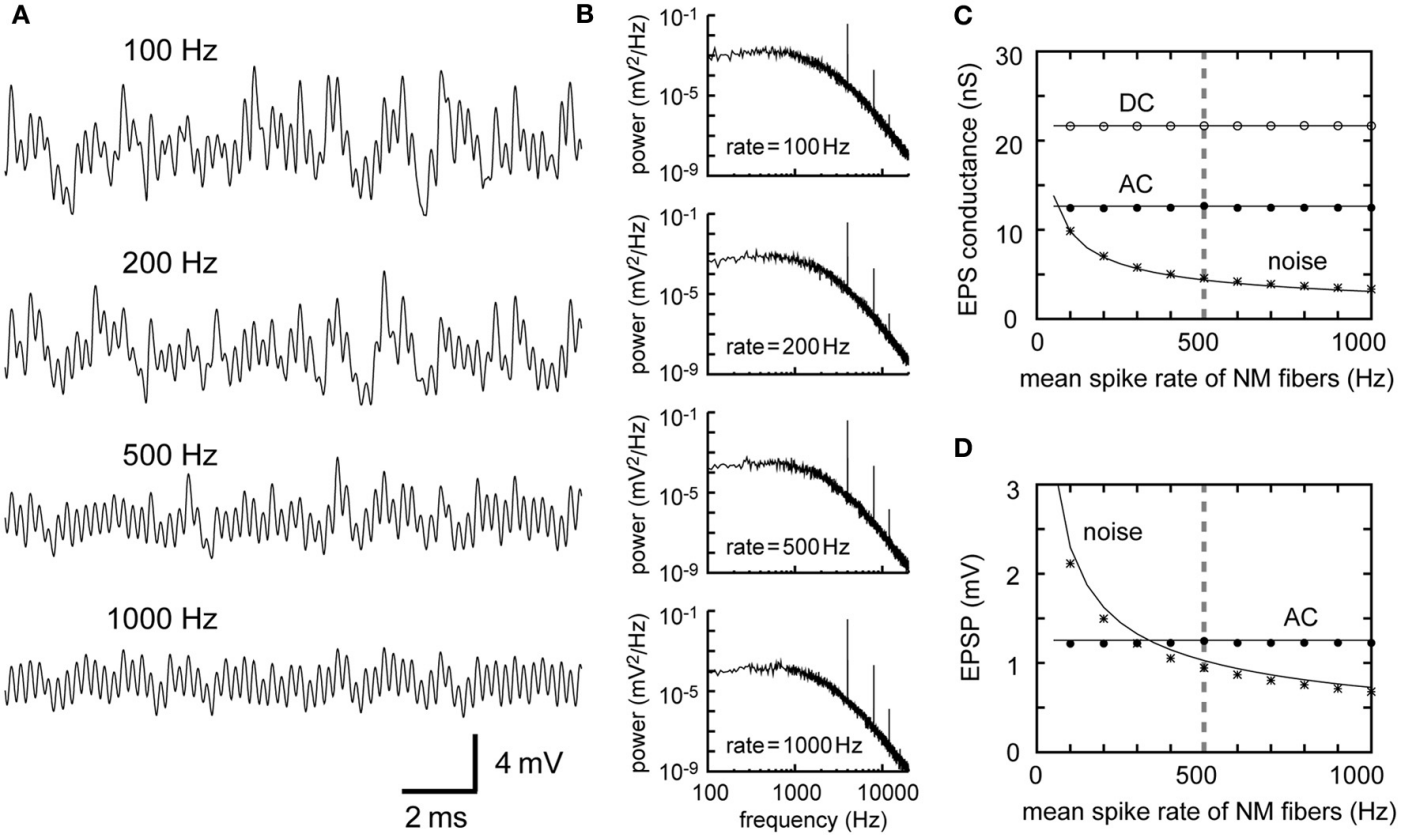
**Dependence of synaptic input in NL on the firing rate of presynaptic NM fibers. (A)** Simulated traces of the model membrane potential. The number above each trace shows the output spike rate of NM fibers. The traces become less noisy as the NM rate increases. **(B)** Power spectral densities of the four traces shown in **(A)**. Low frequency noise components decrease with increasing NM rates, while peaks at the input frequency and higher harmonics remain unchanged. **(C)** Dependence of the DC, AC, and noise amplitudes of the simulated synaptic input on the mean spike rate of NM fibers. **(D)** Dependence of the AC and noise amplitudes of the simulated membrane potential on the mean spike rate of NM fibers. Solid lines in **(C)** and **(D)** are obtained from analytical calculations (Equations 1–5). Vertical broken gray lines in **(C)** and **(D)** show the default parameter (500 Hz) used in our simulations.

The effect of the average NM spike rate λ_0_ is fairly simple. As the linear approximation theory indicates (Equations 1–5 in Table 1), traces of simulated membrane potentials become less noisy as the mean spike rate of presynaptic NM fibers increases (Figure 2A). This observation is confirmed by the power spectral density curves (Figure 2B). Increases in the NM spike rate reduce noise but retain AC and higher harmonics (Figures 2C,D), provided that the average input level (DC) is kept constant. Note that the power spectrum density is discontinuous at frequencies *f* = *nf*_*s*_ (*f*_*s*_: signal frequency; *n* = 0, ±1, ±2,…), corresponding to the DC, AC, and higher harmonics (see Ashida et al., 2013, for further analytical formulations). The noise amplitudes of the synaptic input and the membrane potential decrease linearly with the square root of the spike rate of NM fibers (Equations 3 and 5). The typical spike rate of an NM neuron is over 400 spikes/s (Peña et al., 1996), which is much greater than that of an auditory nerve fiber (Köppl and Yates, 1999). The high spiking rate of NM neurons thus contributes to noise reduction in NL neurons.

### Number of converging NM fibers

**Figure 3:**
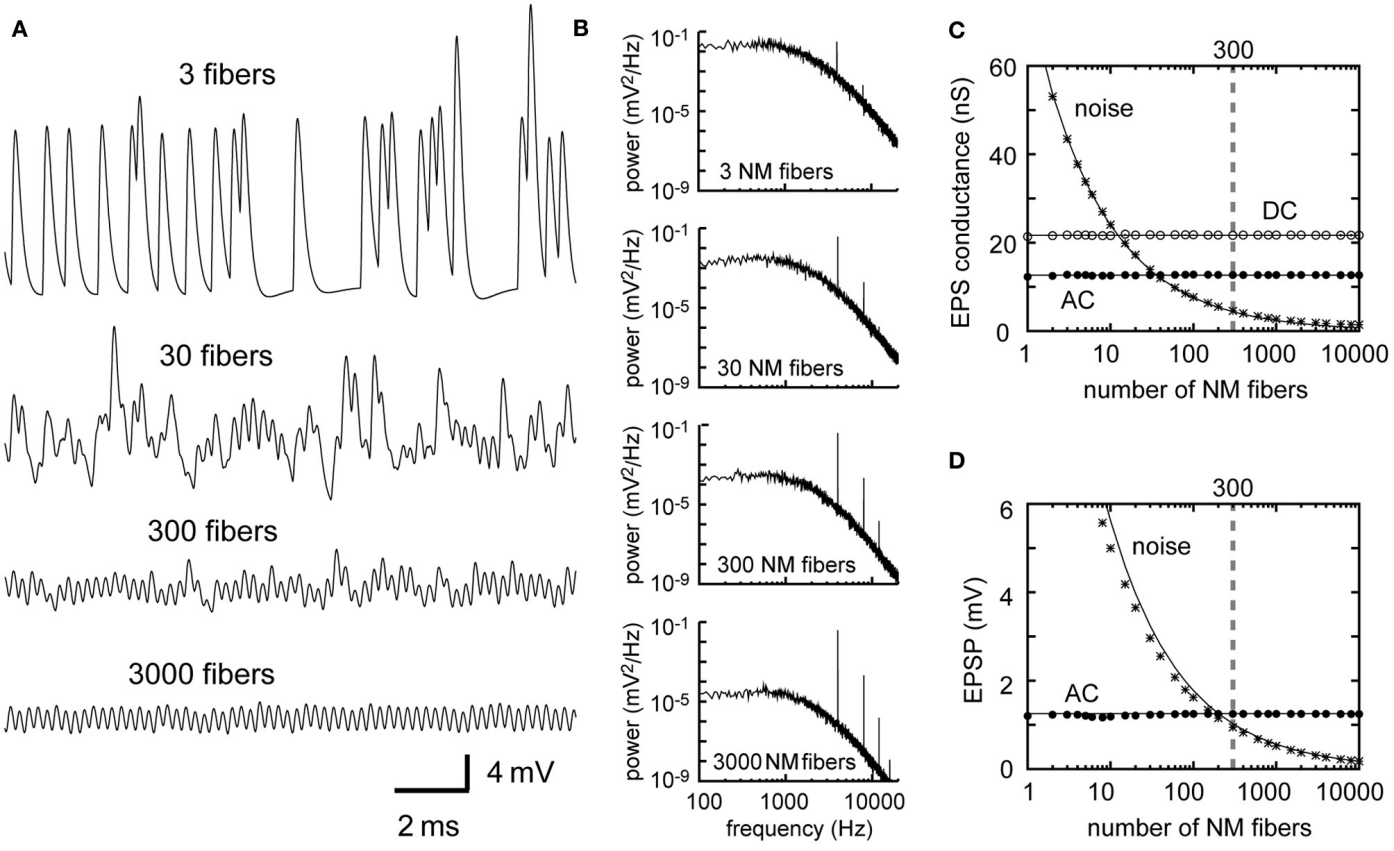
**Dependence of the synaptic input in NL on the number of presynaptic NM fibers. (A)** Simulated traces of the model membrane potential. The number above each trace shows the numbers of NM fibers. The traces become less noisy as the number of NM fibers increases. **(B)** Power spectral densities of the four traces shown in **(A)**. Low frequency noise components decrease with increasing numbers of NM fibers, while peaks at the input frequency and higher harmonics remain unchanged. Although the trace with 3 NM fibers (**A**, top) looks considerably different from the other three traces, its AC component (**B**, top) has the same amplitude as the other three. **(C)** Dependence of the DC, AC, and noise amplitudes of the simulated synaptic input on the number of NM fibers. **(D)** Dependence of the AC and noise amplitudes of the simulated membrane potential on the number of NM fibers. Solid lines in **(C)** and **(D)** are obtained from analytical calculations (Equations 1–5). Vertical broken gray lines in **(C)** and **(D)** show the default parameter (300 fibers) used in our simulations.

The dependence of the AC and noise components on the number *M* of presynaptic fibers is similar to the dependence on the mean spike rate λ_0_ (Equations 2–5 in Table 1). Traces of simulated membrane potential become less noisy as the number M of presynaptic NM fibers increases (Figure 3A). Power spectral density curves support this observation (Figure 3B). The noise amplitude decreases linearly with the square root of *M* (Figures 3C,D), as predicted by the theoretical calculations (Equations 3 and 5). The convergence of large numbers of NM fibers results in stable sinusoidal inputs (Figure 3A, bottom). By contrast, when the number of presynaptic fibers is small, the overall potential waveform is distinct from a pure sinusoid and each unitary synaptic event becomes discernible (Figure 3A, top) even though the calculated AC component itself is the same (Figures 3C,D). An NL cell receives a few hundred NM afferents (Carr and Boudreau, 1993), whereas an NM neuron receives 1–4 auditory nerve inputs via large endbulb synapses (Carr and Boudreau, 1991). Thus, the membrane potential waveforms should be different between NM and NL, potentially reflecting the difference in their computational roles in ITD coding.

### Degree of phase-locking

The degree of phase-locking of the presynaptic NM fibers is quantified by their *VS* (Goldberg and Brown, 1969; Fisher, 1993). The *VS* of a spike sequence at frequency *f* is defined as follows:

$$VS=\frac{1}{N}\sqrt{{\left(\sum_{j\;=\;1}^{N}\cos(2\pi ft_{j})\right)}^{2}+{\left(\sum_{j\;=\;1}^{N}\sin(2\pi ft_{j})\right)}^{2}},$$

where *N* is the total number of spikes in the sequence, and *t*_*j*_ is the timing of the *j*-th spike. Note that the *VS* takes a value between 0 and 1. *VS* = 1 means that all the spikes occurred at a certain phase of the reference frequency *f* (i.e., perfect phase-locking) and a *VS* = 0 implies that the spike sequence has no phase preference.

**Figure 4:**
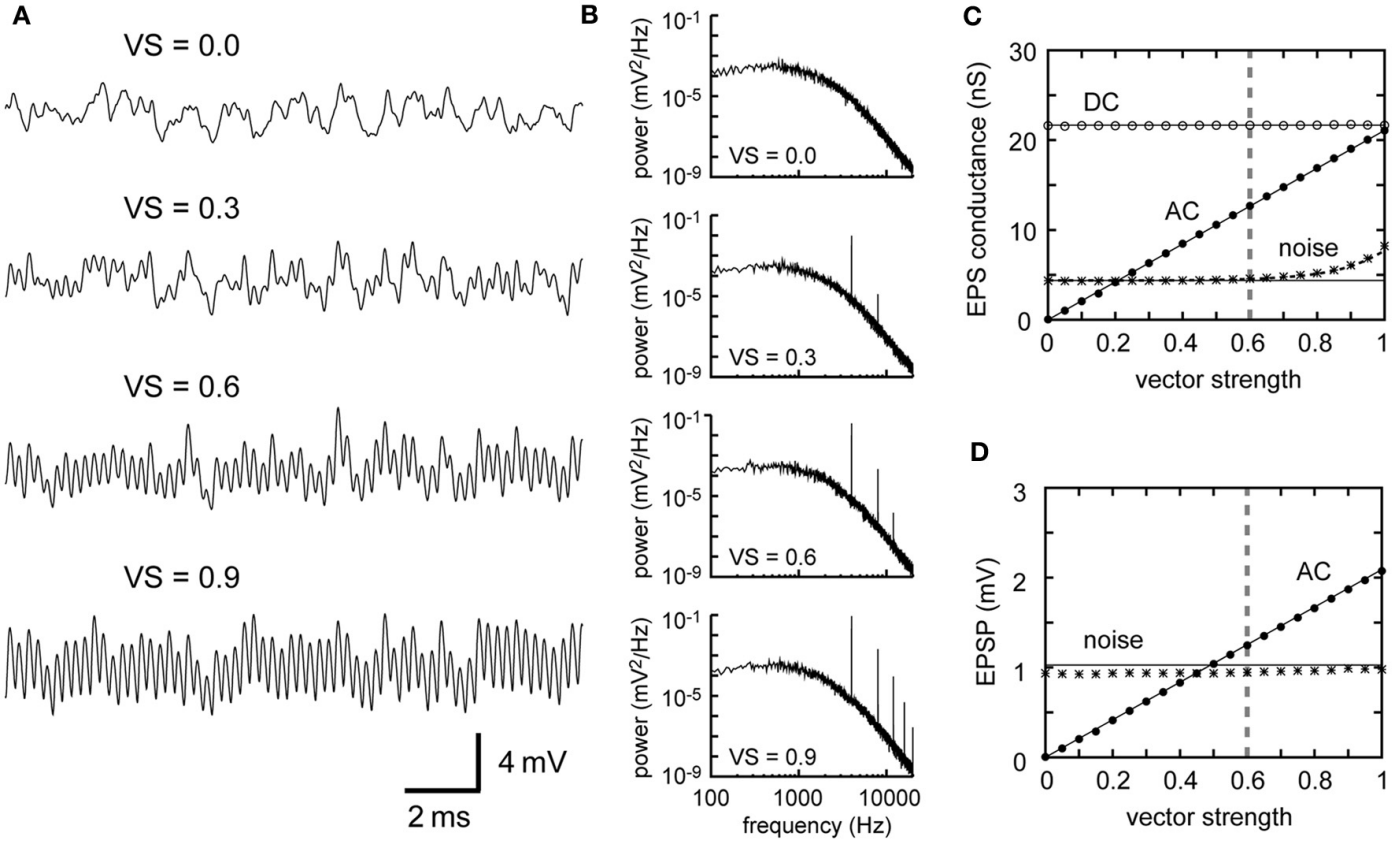
**Dependence of the synaptic input in NL on the degree of phase-locking of presynaptic NM fibers. (A)** Simulated traces of the model membrane potential. The number above each trace shows the vector strength of NM fibers. The traces show larger oscillations (higher AC amplitudes) as *VS* increases. **(B)** Power spectral densities of the four traces shown in **(A)**. Peaks at the input frequency (4 kHz) and higher harmonics increase with increasing *VS*, while the other frequency components remain unchanged. **(C)** Dependence of the DC, AC, and noise amplitudes of the simulated synaptic input on *VS*. **(D)** Dependence of the AC and noise amplitudes of the simulated membrane potential on *VS*. Solid lines in **(C)** and **(D)** are obtained from analytical calculation without higher harmonics included. The dotted black line in **(C)** is obtained from analytical calculations with the second harmonic included. Vertical broken gray lines in **(C)** and **(D)** show the default parameter (*VS* = 0.6) used in our simulations.

Increases in the *VS* of the NM inputs lead to a gain in the AC component without altering the noise (Figures 4A,B). Theoretical calculations (Equations 2 and 4) and simulations (Figures 4C,D) indicate a linear relationship between AC and *VS*. This can be explained as follows: *VS* can be calculated as the absolute value of the Fourier component of the spike sequence at the stimulus frequency, normalized by the total number of spikes, regarding each spike as a delta function (Ashida et al., 2010). Within the regime where the synaptic and the membrane processes act as linear filters, the Fourier components of the resulting synaptic conductance and the membrane potential at the stimulus frequency are still linear with the *VS*. Consequently, the AC component, which is linearly related to the Fourier component, is linear to the *VS*. The prominent phase-locking property of NM fibers up to 8 kHz (Sullivan and Konishi, 1984; Köppl, 1997) is thus a fundamental component of high frequency ITD coding of the barn owl.

Higher harmonics, which are regarded as part of the noise, also increase with *VS* (Figure 4B). Hence the noise amplitude in the input conductance is no longer constant for *VS* > 0.7 (Figure 4C). The noise component in the membrane potential, however, is almost independent of *VS* (Figure 4D), because the low-pass properties of the membrane effectively filter out higher harmonics of 8 kHz and above (see accompanying paper: Ashida et al., 2013, for how higher harmonics change with *VS*). It should be noted that even when all the input spikes are perfectly phase-locked (i.e., *VS* = 1), noise does not disappear (Figures 4C,D). This is due to the cycle-to-cycle variability. With our default parameters (*M* = 300 fibers spiking at λ_0_ = 500 Hz locked to *f*_*s*_ = 4 kHz tonal stimulus), for example, the probability *P* that each fiber spikes at a certain stimulus cycle can be calculated as *P* = λ_0_/*f*_*s*_ = 500/4000 = 0.125. Then the average number of inputs counted in a single stimulus cycle is *MP* = 37.5, with a standard deviation being $\sqrt{MP(1-P)}=5.73$. Therefore, even with perfect phase-locking, cycle-to-cycle variability of spike count would be on the order of 5.73/37.5 = 15%.

### Synaptic time constant

**Figure 5:**
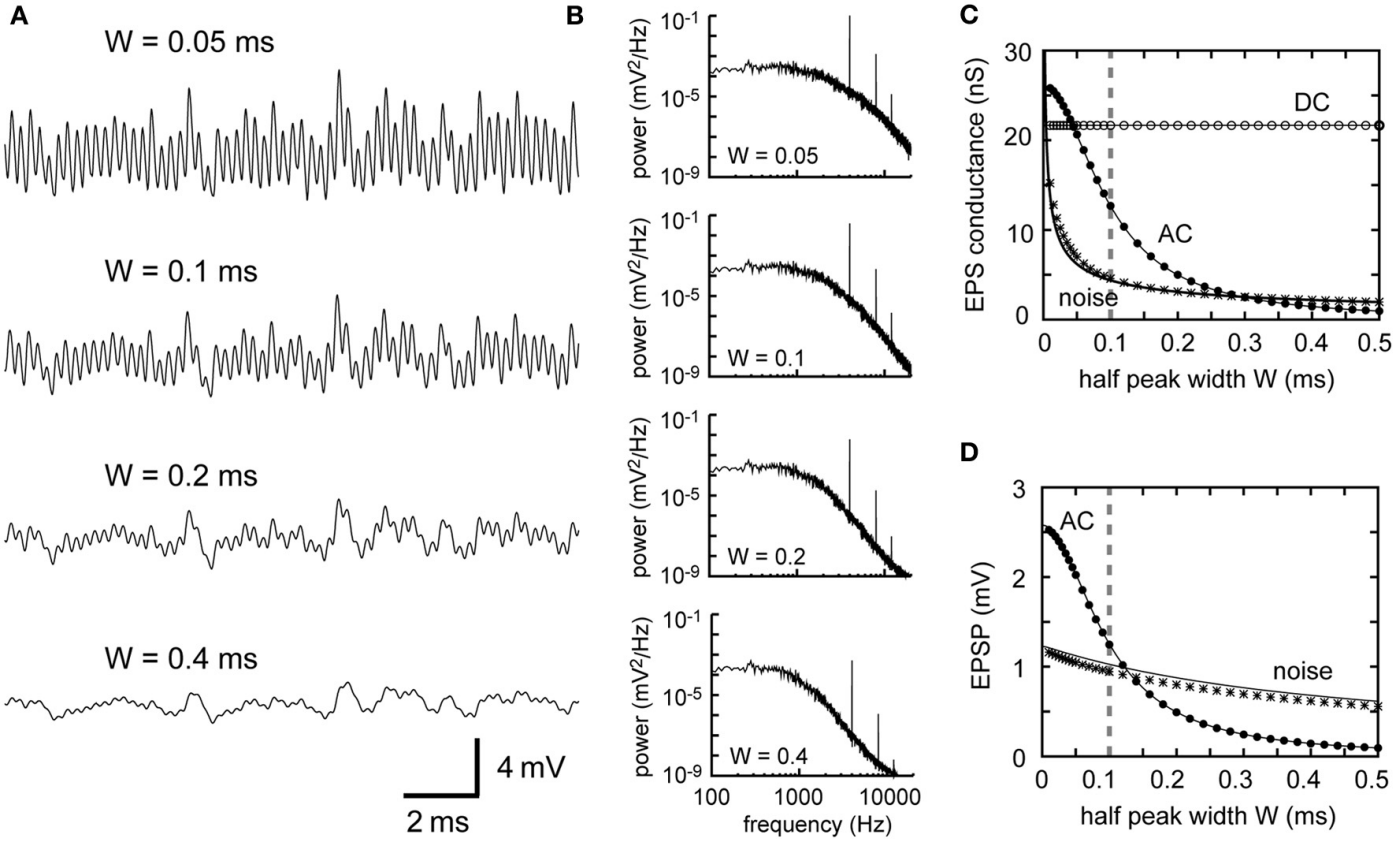
**Dependence of synaptic input in NL on the synaptic time scale. (A)** Simulated traces of the model membrane potential. The number above each trace shows the half peak widths *W* of the unitary synaptic input modeled by an alpha function (see Table 1). The traces show larger oscillations (higher AC amplitudes) as W decreases. **(B)** Power spectral densities of the four traces shown in **(A)**. Higher frequency components decrease with increasing *W*. **(C)** Dependence of the DC, AC, and noise amplitudes of the simulated synaptic input on the synaptic time scale *W*. **(D)** Dependence of the AC and noise amplitudes of the simulated membrane potential on the synaptic time scale W. Solid lines in **(C)** and **(D)** are obtained from analytical calculations (Equations 1–5). Vertical broken gray lines in **(C)** and **(D)** show the default parameter (*W* = 0.1) used in our simulations.

**Figure 6:**
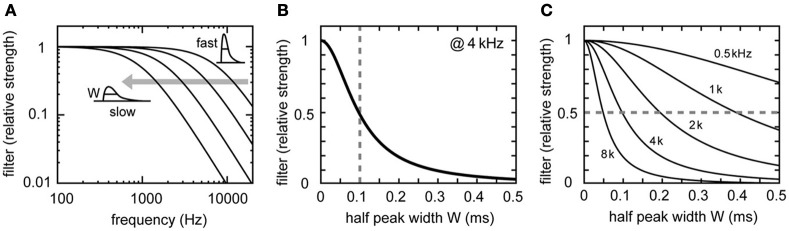
**Frequency properties of the synaptic filter. (A)** Fourier transform |*F*_α_(*f*)| of the synaptic filter α(*t*) (see Table 1). Normalized curves with *W* = 0.05, 0.10, 0.20, and 0.40 are shown. The synaptic filter becomes more likely to reduce high frequency components as the synaptic time scale *W* becomes smaller. **(B)** Synaptic filter at 4 kHz showing non-linear dependence on *W*. The vertical broken gray line in **(B)** shows the default parameter (*W* = 0.1) used in our simulations. **(C)** Comparison of synaptic filters at different sound stimulus frequencies *f*_*s*_ (0.5, 1, 2, 4, and 8 kHz). Filter strength exceeds 0.5 (broken gray line), if and only if the inequality *W* < *k*/(2π*f*_*s*_) is satisfied. Note that this critical *W*-value is dependent on the frequency.

Changes in the synaptic time scale (measured by the half peak width *W* of the unitary synaptic input) affect both the AC and noise responses (Figures 5A,B). Changes in *W* shift the filtering property of the synaptic input (Figure 6A). Slowing down the synaptic process reduces high frequency components. To conserve the 4 kHz signal component, the half peak width *W* should be equal to or smaller than the order of 0.1 ms (Figures 5C,D, 6B; see Funabiki et al., 2011, for related discussion). Decrease in *W* (i.e., speeding up the synaptic process) generally results in an increase in the AC and noise components of the input conductance (Figure 5C). As Equation 3 indicates, the noise component of the synaptic conductance blows up to infinity with *W* approaching to zero (Figure 5C). In the membrane potential (Figure 5D), however, the increase in noise with decreasing *W* is much slower because the increasing noise consists mostly of high frequency components (Figure 6A), which are filtered out by the low-pass effects of the membrane. As a result, the AC is more sensitive than the noise to the change in the width *W* of the unitary synaptic input (Figure 5D).

The half peak width *W* of the unitary synaptic input is linear to the time constant τ of the alpha function (Table 1). The AC component of the input conductance *A*_*G*_ = (2*rD*_*G*_)/(1 + (2π*f*_*s*_τ)^2^) (see accompanying paper, Ashida et al., 2013, for detailed derivations). It is therefore necessary that *W* < *k*/(2π*f*_*s*_) in order to retain an effective amount of AC input (Figure 6C). This is roughly equivalent to *W* < 0.4/*f*_*s*_, with *f*_*s*_ being the stimulus sound frequency. If *W* is larger than this criterion, the AC component is effectively filtered out by the synaptic process (e.g., Figure 5A, *W* = 0.4 ms). To reproduce the sound analog potential observed *in vivo* (AC = 1–2 mV, Funabiki et al., 2011), the synaptic time scale should be at least several times faster than that observed in chick high frequency NL cells *in vitro* (*W* = 0.2–0.3 ms, frequency range = 2.5–3.3 kHz, Kuba et al., 2005).

### Implications for ITD coding

**Figure 7:**
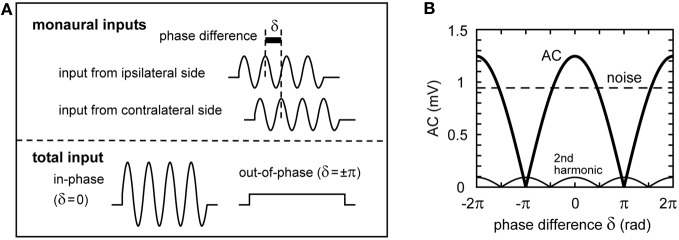
**Schematic representation of the binaural synaptic input to the NL neuron. (A)** Summation of the synaptic input from ipsi- and contralateral NM fibers. The oscillation amplitude of the total synaptic input in NL is maximal when the two inputs arrive perfectly in-phase, while it becomes smaller when the two inputs are out of phase. For clarity, onset effects, higher harmonics, and noise components are not included in this schematic figure. **(B)** The oscillation amplitude of the total NM inputs. The amplitude of the AC component changes periodically with the phase difference δ of the two inputs according to the Equation: Amp(δ) = |*A* cos(δ/2)|, with *A* being the maximum AC amplitude (Ashida et al., 2007). The second harmonic also changes periodically with δ, but its period is half of that of the main signal (AC).

In the preceding sections, we investigated the dependence of the sound analog membrane potential on the parameters of the phase-locked synaptic inputs. In this section, we examine how these parameters affect ITD coding. In the avian auditory brainstem, ITDs are compensated by the NM-axonal delay lines and computed by the coincidence detector neurons in NL (Carr and Konishi, 1990; Köppl and Carr, 2008). The phase difference δ between the bilateral synaptic inputs from NM to NL reflects the ITD (Figure 7A; see Funabiki et al., 2011, for more discussion). The AC component (4 kHz) of the total synaptic input changes periodically with the phase difference δ (Figures 7A,B), while the noise component is independent of δ (Figure 7B). The second harmonic (8 kHz), whose period is half of the main AC signal, is less than 0.1 mV and shows different dependence on the phase δ (Figure 7B).

**Figure 8:**
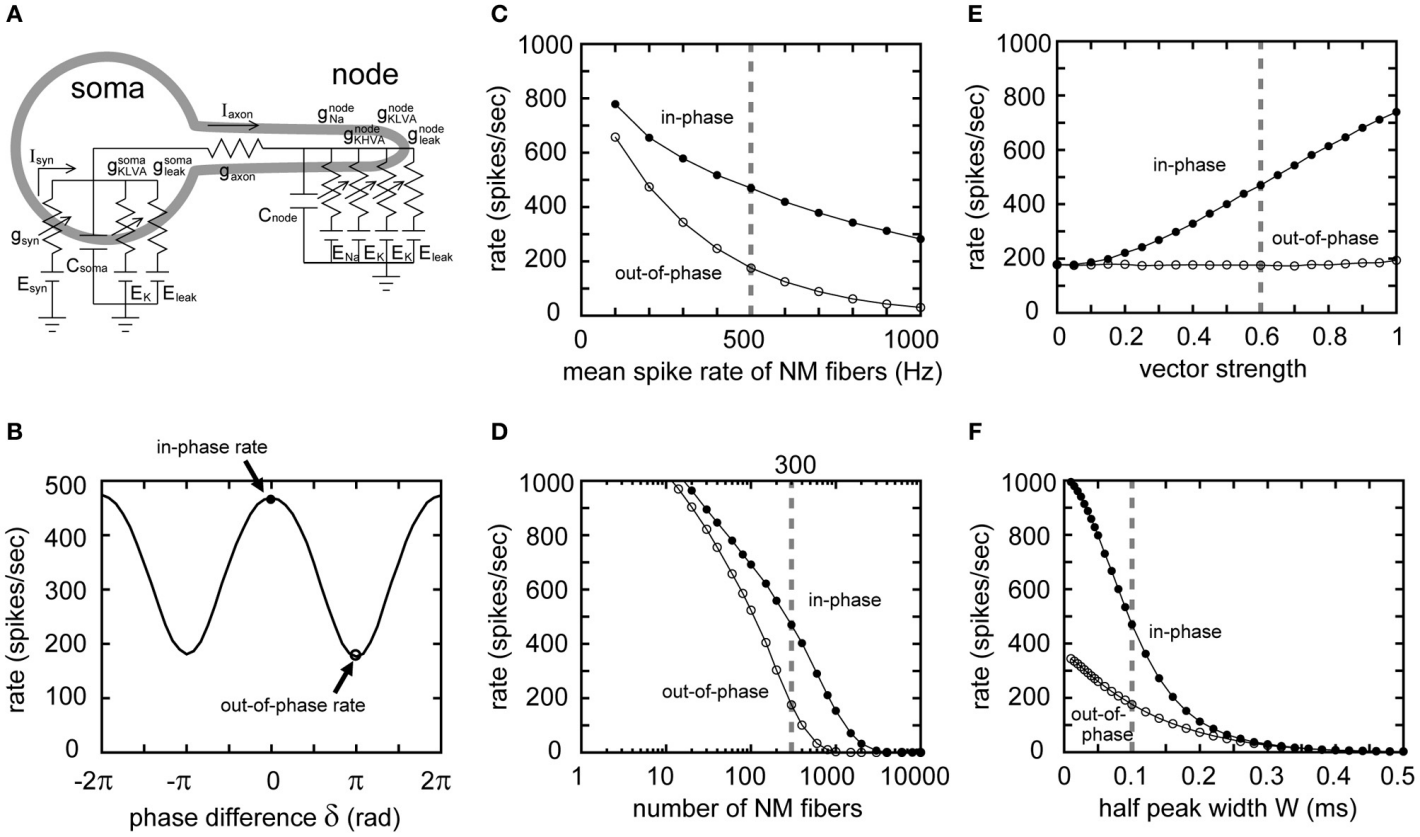
**Reponses of the two-compartment NL model to 4 kHz input. (A)** Two-compartment NL neuron model, with a soma and a node interconnected by an axonal conductance. The somatic compartment has the same amount of leak and low-voltage-activated potassium (K_LVA_) conductances as the single compartment model. In addition to the leak and K_LVA_, the nodal compartment has Na and high-voltage-activated potassium (K_HVA_) conductances to generate spikes. **(B)** Spike rate of the model neuron plotted against the phase difference δ between ipsi- and contralateral model NM inputs. We define the spike rate with δ = 0 as the “in-phase rate,” and δ = ±π as the “out-of-phase rate.” In-phase and out-of-phase rates are plotted against the mean spike rate of NM fibers **(C)**, the total number of NM fibers **(D)**, the degree of phase-locking of the NM fibers **(E)**, and the half peak width *W* of the unitary synaptic input conductance **(F)**. Vertical broken gray lines in **(C–F)** show the default parameters (λ_0_ = 500 Hz, *M* = 300 fibers, *r* = 0.6, *W* = 0.1 ms) used in our simulations.

In the previous sections, and in our accompanying paper (Ashida et al., 2013), we used a single compartment NL model without a spike generator to focus on the fundamental properties of the sound analog membrane potential. In this section, however, we use a two-compartment NL model (Ashida et al., 2007; Funabiki et al., 2011) in which the cell body (soma) receives synaptic input and the axonal node generates spikes (Figure 8A), to study how sound analog potentials are converted to the output spike rate of the NL neuron. With the default input parameters, the spike rate of the model neuron is modulated periodically with the phase δ of the ipsi- and contralateral inputs, indicating a linear conversion of the AC signals into spike rate (Figure 8B; see Funabiki et al., 2011, for detailed discussion on the linear conversion). Generally, the model neuron shows the highest spike rate when the two inputs arrive perfectly in-phase (δ = 0 in Figure 8B). We hereafter refer to this maximum spike rate as the “in-phase rate.” When the inputs arrive in perfect anti-phase (δ = ±π in Figure 8B), the spike rate becomes lowest, which we call the “out-of-phase rate.” Note that the out-of-phase rate is the spike rate driven primarily by noise, while the in-phase rate is driven by both AC and noise components.

Varying the mean spike rate (λ_0_) of NM, the number (*M*) of NM fibers, the degree (*r*) of phase-locking of these fibers, and the synaptic time scale (*W*), we calculated the in-phase and out-of-phase rates of the model neuron. The out-of-phase rate increases with both decreasing NM spike rate (Figure 8C) and decreasing numbers of NM inputs (Figure 8D), because of the increasing noise (Figures 2D, 3D). The in-phase-rate is higher than the out-of-phase rate by a few hundred Hz (Figures 8C,D), provided that the number of NM fibers exceeds 100 and their spike rates are over a few hundred Hz. Changes in *VS* do not alter the out-of-phase rate (Figure 8E) because the noise component without higher harmonics is independent of *VS* (Figure 4D). The out-of-phase curve is flat even near *VS* = 1 (Figure 8E), indicating a small contribution of the second and higher harmonics in our 4 kHz NL model (Figure 7B). The in-phase rate increases almost linearly with *VS* (Figure 8E), in agreement with the linear conversion of the AC component to spike rate found in NL neurons *in vivo* (Funabiki et al., 2011). Since both the AC and noise components increase with decreasing synaptic time scale *W* (Figure 5D), both the in-phase and out-of-phase rates also increase with decreasing *W* (Figure 8F). The increase in the in-phase-rate is, however, more prominent, because *W* is more likely to affect the AC amplitude than the noise amplitude (Figure 5D). Therefore, the modulation depth, which is the difference between the in-phase and out-of-phase rates, becomes higher for smaller *W* (i.e., faster synaptic inputs). In our simulation, *W* needed to be equal or smaller than 0.1 milliseconds for the 4 kHz NL model neuron to obtain modulation depths of a few hundred Hz, consistent with measured spike rates in barn owl's NL (Peña et al., 1996; Funabiki et al., 2011).

## Discussion

Phase-locking, or the generation of action potentials at a certain phase of the reference signal, contributes to fine temporal information coding in the auditory system (Oertel, 1999; Ashida et al., 2010; Brette, 2012). In the owl's auditory brainstem, phase-locked synaptic input sequences from the NM axons are filtered by synaptic and membrane processes, leading to oscillatory membrane potentials in NL (Gerstner et al., 1996; Kempter et al., 1998; Ashida et al., 2007; Funabiki et al., 2011). The phase differences between inputs from ipsi- and contralateral NM, which are the consequence of ITD, lead to the periodic changes in the AC component of the binaural input (Figure 7). The NL neuron acts as a linear converter of AC signals into spike rate (Funabiki et al., 2011), and thus changes spike rate periodically with ITD or with the input phase difference (Figure 8B). Similar oscillatory membrane potentials were found in auditory hair cells (Russell and Sellick, 1978; Dallos, 1985). Unlike auditory hair cells, however, NL neurons are located a few synapses away from the cochlea. The sound analog potential in NL is therefore affected by various presynaptic, synaptic, and postsynaptic factors.

### Model assumptions

In this paper, we focused particularly on the sound analog membrane potential induced by pure tones. Since the NL neuron is narrowly tuned to frequency (Peña et al., 2001), the input to each NL neuron is expected to be dominated by the AC component at or near its characteristic frequency even if the stimulus sound is broadband. Nevertheless, for more complex stimuli than simple tones, multiple frequency components might affect ITD coding in more complicated fashion. For example, different frequency components show different dependence on the synaptic time constant (Figure 6C). Thus, the optimal synaptic time scale for pure tones could differ from that for broadband stimuli. More modeling and physiological investigation will be necessary to examine how complex stimuli is presented and processed in owls' NL.

In our series of numerical simulations, we fixed the average input level (DC) in order to focus on the effects of the AC and noise components on the ITD coding performance of the model NL neuron. This assumption is based on the following observations: (1) the DC component is irrelevant to ITD coding (Funabiki et al., 2011); (2) the NL neuron increases its spike rate even with a small amount of external current injection (Funabiki et al., 2011); and (3) the DC level should be carefully chosen so that the model neuron shows good sensitivity to AC signals (Ashida et al., 2007). For these reasons, we presumed that the DC input of the NL neuron *in vivo* should be optimized to efficiently compute ITD-dependent AC signals. In our simulations we determined the DC level so that the difference between the in-phase and out-of-phase spike rates exceeded 180 spikes/s (Figure 8B), as was observed *in vivo* (Peña et al., 1996). Large increases or decreases in the constant DC should lead to over- or under-excitability of the neuron, resulting in the degradation of the overall ITD coding performance (see also Funabiki et al., 2011, for related discussion on the effects of DC).

### Presynaptic factors

Changes in both the number (Figure 2) and spike rate (Figure 3) of presynaptic NM fibers alter the noise component but not the AC signal. In the ITD coding pathway of the barn owl, an NM neuron receives 1–4 auditory nerve terminals (Carr and Boudreau, 1991), while an NL neuron is estimated to receive a few hundred NM afferents (Carr and Boudreau, 1993) and a neuron in the anterior part of the dorsal lateral lemniscus (LLDa) is estimated to receive 2–10 NL inputs (Fischer and Konishi, 2008). The primary role of the NM neuron is to convey precise temporal information from auditory nerves to NL (Sullivan and Konishi, 1984; Köppl, 1997), whereas coincidence detector neurons in NL compare bilateral NM inputs using the sound analog potentials, whose amplitudes systematically change with ITD (Funabiki et al., 2011). The LLDa provides finer ITD tuning than NL by reducing noise (Fischer and Konishi, 2008). The optimal convergence number of input fibers for 4 kHz ITD computation in NL is predicted to be on the order of a few hundred (Figure 8D), whereas the optimal number for NM and LLDa, which have different computational roles, should be smaller than NL.

In contrast to the owl's NL neurons, neurons in the gerbil's MSO, the mammalian counterpart of NL, have recently been reported to receive less than 10 excitatory synaptic inputs (Couchman et al., 2010). Thus, the membrane potential of the MSO neuron may not show the clear sinusoidal waveforms that characterize responses in the owl's NL. Whereas the NL neuron receives only slow GABAergic inhibition (Burger et al., 2011; Coleman et al., 2011), the gerbil's MSO neurons receive fast glycinergic inhibition (Magnusson et al., 2005), which plays an essential role in the ITD coding in the MSO (Brand et al., 2002; Pecka et al., 2008). The difference in computational strategies in MSO and NL may include differences in the number of inputs in these neurons.

The degree of phase-locking, as measured by *VS*, has a linear relationship to the resulting AC signal (Figure 4), but does not affect the noise, which originates from the cycle-to-cycle variability of input spike counts. The in-phase rate, therefore, monotonically increases with *VS*, while the out-of-phase rate remains almost independent of *VS* (Figure 8E). Thus, the prominent phase-locking ability observed in owl's NM (Köppl, 1997) leads directly to the high computational efficiency in NL. In the accompanying paper (Ashida et al., 2013), we pointed out that perfect phase-locking may not always be beneficial to ITD coding because of the detrimental effects of higher harmonics. In our 4 kHz model neuron, however, the amplitude of the second harmonic is less than 10% of the signal amplitude (Figure 7B) because of the low-pass property of the membrane (compare Figures 4C,D). Thus, the negative contribution higher harmonics should be limited to NL neurons with best frequencies of 2 kHz or below.

### Synaptic factors

Short synaptic time constants are one of the most important features of high-frequency coincidence detection. Auditory coincidence detector neurons in the chicken NL (Kuba et al., 2005) and gerbil MSO (Couchman et al., 2010) receive extremely fast synaptic inputs with time constants of far below a millisecond. The synaptic time scale, measured by the half peak width of unitary synaptic input (*W*), affects both AC and noise components. Although these components of the input conductance increase with decreasing synaptic time constant (Figure 5C), the effect of the increasing noise on the membrane potential is smaller than that of the AC component (Figure 5D), because the membrane filter cuts out high frequency noise that appears with decreasing *W* (Figure 6A). In consequence, the modulation between in-phase and out-of phase rates becomes higher for smaller *W* (Figure 8F). In order to obtain a sufficient AC signal, *W* needs to be smaller than 0.4/*f*_*s*_ (ms), with *f*_*s*_ being the signal frequency (in kHz). This implies that the higher the cell's signal frequency, the faster the synaptic input process should be (Slee et al., 2010). Previous studies of NL (Grau-Serrat et al., 2003) and mammalian MSO (Mathews et al., 2010; Khurana et al., 2011) identified various mechanisms underlying the submillisecond accuracy of synaptic inputs. Jercog et al. (2010) showed that synaptic inputs from the ipsilateral side are faster than those from the contralateral side, contributing to the fine ITD tuning of MSO neurons. Further investigation is necessary to determine how fast the actual owl's NL synapses are *in vivo*.

### Postsynaptic factors

Membrane time constants of about 0.1 ms or less are essential for high frequency signal processing in NL (Gerstner et al., 1996; Kempter et al., 1998). Impedance analyses (Gutfreund et al., 1995; Ashida et al., 2007) showed that a neuron that handles high frequency signals needs to be both leaky (i.e., having a low input resistance) and electrotonically compact (i.e., having a small capacitance). Short dendrites (Carr and Konishi, 1990; Carr and Boudreau, 1993; Agmon-Snir et al., 1998; Kuokkanen et al., 2010) and large K_LVA_ conductances (Kuba et al., 2005) are consistent with these requirements. It should be noted that even with membrane properties that allow high frequency signals up to several kHz, the membrane still acts as a low-pass filter (see accompanying paper: Ashida et al., 2013) and efficiently eliminates higher harmonics (Figures 4C,D, 5C,D, 7B). Recent simulation results suggest that the kinetic properties of the K_LVA_, such as increased activation with depolarization, may improve ITD computation in the gerbil's MSO neuron (Svirskis et al., 2002; Day et al., 2008; Gai et al., 2009; Jercog et al., 2010; Mathews et al., 2010). Dynamic interplay between K_LVA_ and hyperpolarization-activated cation currents has been suggested to stabilize synaptic inputs in the MSO (Khurana et al., 2011). How these conductances dynamically affect the computation of high frequency AC signals in owl's NL remains to be elucidated.

### Other factors

In the owl's NL, ITD tuning has been found up to about 7 kHz (Carr and Konishi, 1990). In our preliminary simulations, however, it was hard to reproduce ITD tuning at 7 kHz (data not shown), primarily due to the decrease in AC amplitude with increasing frequency (see also Figure 4 of our accompanying paper: Ashida et al., 2013). Therefore, additional mechanisms and finer tuning should be incorporated in the actual ITD coding of the barn owl. Given that *VS* decreases with frequency (Köppl, 1997), high-frequency NL neurons would require more inputs to reduce noise (Figure 3D), as well as faster synaptic inputs (Figure 5D) and smaller membrane time constants (Kempter et al., 1998) to preserve AC signals. In our modeling, we focused on the NM-NL circuit, where only excitatory synaptic inputs were considered. In the avian brainstem, however, both NM and NL neurons receive GABAergic inhibitory inputs from the superior olivary nucleus (Carr et al., 1989; Burger et al., 2005; Coleman et al., 2011; see Burger et al., 2011, for a recent review), which play an important role in refining ITD tuning in NL (Fujita and Konishi, 1991; Funabiki et al., 1998; Yang et al., 1999; Nishino et al., 2008). Inhibitory inputs might contribute in stabilizing the formation of sound analog potential.

In addition, neuronal activity in the Mauthner cell cap (Korn and Faber, 1975), hippocampus (Radman et al., 2007), and in the cortex (Anastassiou et al., 2011) can be affected by inhomogeneous distributions of the extracellular field potential. This effect, called the ephaptic coupling, provides a strong basis of synchronous spike activities in these brain areas. In the owl's NL, the neurophonic, or the extracellular field potential, is strongly correlated with tonal stimuli whose amplitudes are in the millivolt range (see Kuokkanen et al., 2010; and references therein). If ephaptic coupling occurs between the neurophonic and NL neurons, it could be an additional mechanism of mediating the sound analog potential.

## Materials and methods

The detailed modeling procedure and its analytical consideration are provided in our previous (Ashida et al., 2007; Funabiki et al., 2011) and accompanying (Ashida et al., 2013) papers. Here we summarize the fundamental equations of our model NM-NL system.

### Modeling phase-locked synaptic input to NL

In order to model the phase-locked NM activity, we used an inhomogeneous Poisson process with a time-dependent intensity function λ(*t*) = 2πλ_0_ *p*_κ_(2π*f*_*s*_*t*), where λ_0_ is the average intensity, *p*_κ_ is the von-Mises distribution function (parameterized by the concentration parameter κ) and *f*_*s*_ is the stimulus sound frequency. The model NL neuron receives inputs from *M* fibers that are phase-locked to the stimulus tone with a *VS* of *r* = *I*_1_(κ)/*I*_0_(κ), with *I*_*n*_ being the Modified Bessel function of order *n*. In order to model the unitary synaptic conductance in NL, we used an alpha-function α(*t*) = (*Ht*/τ) exp(1 − *t*/τ), with *H* being the peak height and τ being the time constant. Note that the half peak width *W* of the alpha function is linearly related to τ as *W* = 2.446τ All the synaptic conductance input is linearly summed and delivered to the soma of the model NL neuron. The model equations and parameters used in this paper are summarized in Table 1.

In the present paper, we changed the average spike rate of NM fibers (λ_0_), the number of converging fibers to the model NL neuron (*M*), the average degree of phase-locking (*r*) of the NM fibers, and the synaptic time scale (*W*). In numerical simulations where λ_0_, *M*, or *W* was changed, the peak height *H* of the unitary synaptic input was re-scaled to conserve the total input conductance. For example, when the mean spike rate of NM fibers was set at twice (*M* = 1000 Hz) the default rate (*M* = 500 Hz), the peak height *H* was reduced to the half (0.65 nS) of the default value (1.3 nS).

### Single compartment NL neuron model

In order to examine the parameter dependence of the AC and noise components of the synaptic input and the membrane potential of the model NL neuron, we used a conductance-based single compartment model. The dynamics of the membrane potential of the soma *V*_*S*_ is described by a first-order differential Equation:

$${\text{C}}_{\text{S}}\frac{d}{dt}V_{S}(t)=I_{L}{}^{S}+I_{\text{KLVA}}{}^{S}+I_{\text{axon}}{}^{S}+I_{\text{syn}},$$

where *C*_*S*_ is the membrane capacitance, *I*_*L*_^*S*^ = *g*_*L*_^*S*^(*E*_*L*_ − *V*_*S*_) is the leak current, *I*_KLVA_^*S*^ = *ḡ*_KLVA_^*S*^ *d*(*V*_*S*_, *t*)(*E*_*K*_ − *V*_*S*_) is the K_LVA_ current, *I*_axon_^*S*^ is the axonal current, and *I*_syn_ = *g*_syn_(*E*_syn_ − *V*_*S*_) is the model synaptic input. For the single compartment model, the axonal current was fixed to zero. The activation variable *d*(*V, t*) of the K_LVA_ conductance obeys the first-order differential Equation:

$$\tau_{d}\frac{d}{dt}d(V,t)=-d(V,t)+d_{\infty }(V).$$

Membrane properties, including the maximum K_LVA_ conductance *ḡ*_KLVA_, were fixed in our simulations.

### ITD dependence and two-compartment NL neuron model

A two-compartment NL neuron model was used to examine the parameter dependence of output spike rate (see Ashida et al., 2007; for detailed explanations for the two-compartment model). The model consists of the soma and the node connected by the axonal resistance (Figure 8A). The model equations and parameters are the same as in our previous study (Funabiki et al., 2011). The somatic variables and parameters are the same as those used for the single compartment model. Sodium and high-voltage-activated potassium (K_HVA_) conductances were introduced in the nodal compartment to generate spikes. The membrane potential of the node *V*_*N*_ is described as:

$$C_{N}\frac{d}{dt}V_{N}(t)=I_{L}{}^{N}+I_{\text{KLVA}}{}^{N}+I_{\text{HLVA}}{}^{N}+I_{\text{Na}}{}^{N}+I_{\text{axon}}{}^{N},$$

where *I*_*L*_^*N*^ = *g*_*L*_^*N*^(*E*_*L*_ − *V*_*N*_) is the leak current, *I*_KLVA_^*N*^ = *ḡ*_KLVA_^*N*^ *d*(*V*_*N*_, *t*)(*E*_*K*_ − *V*_*N*_) and *I*_KHVA_^*N*^ = *ḡ*_KLVA_^*N*^ *n*(*V*_*N*_, *t*)(*E*_*K*_ − *V*_*N*_) are, respectively, the K_LVA_ and K_HVA_ currents, *I*_Na_^*N*^ = *ḡ*_Na_^*N*^ *m*(*V*_*N*_, *t*)*h*(*V*_*N*_, *t*)(*E*_Na_ − *V*_*N*_) is the fast sodium current, and *I*_axon_^*S*^ = −*I*_axon_^*N*^ = *g*_axon_(*V*_*N*_ − *V*_*S*_) is the axonal current. The K_HVA_ conductance has only the activation variable *n*(*V, t*), while the sodium conductance has both activation *m*(*V, t*) and inactivation *h*(*V, t*) variables. These variables obey the equation $\tau_{x}\frac{d}{dt}x(V,t)=-x(V,t)+x_{\infty }(V)$, where *x* stands for *n, m*, or *h*. The model equations and parameters used in this paper are summarized in Table 2.

**Table 2:** **Equations and parameters of the two-compartment NL model**.

**Variable/parameter**	**Equation/value**
K_LVA_ channel activation *d*(*V, t*)	τ_*d*_(*V*) = *Q*^(*T* − 23)/10^_10_/(α_*d*_(*V*) + β_*d*_(*V*))
	*d*_∞_(*V*) = α_*d*_(*V*)/(α_*d*_(*V*) + β_*d*_(*V*))
	α_*d*_(*V*) = 0.20 exp((*V* + 60)/21.8)
	β_*d*_(*V*) = 0.17 exp(−(*V* + 60)/14)
K_HVA_ channel activation *n*(*V, t*)	τ_*n*_(*V*) = *Q*^(*T* − 23)/10^_10_/(α_*n*_(*V*) + β_*n*_(*V*))
	*n*_∞_(*V*) = α_*n*_(*V*)/(α_*n*_(*V*) + β_*n*_(*V*))
	α_*n*_(*V*) = 0.110 exp((*V* + 19)/9.1)
	β_*n*_(*V*) = 0.103 exp(−(*V* + 19)/20)
Na channel activation *m*(*V, t*)	τ_*m*_(*V*) = *Q*^(*T* − 23)/10^_10_/(α_*m*_(*V*) + β_*m*_(*V*))
	*m*_∞_(*V*) = α_*m*_(*V*)/(α_*m*_(*V*) + β_*m*_(*V*))
	α_*m*_(*V*) = 3.6 exp((*V* + 34)/7.5)
	β_*m*_(*V*) = 3.6 exp(−(*V* + 34)/10.0)
Na channel inactivation *h*(*V, t*)	τ_*h*_(*V*) = *Q*^(*T* − 23)/10^_10_/(α_*h*_(*V*) + β_*h*_(*V*))
	*h*_∞_(*V*) = α_*h*_(*V*)/(α_*h*_*V* + β_*h*_(*V*))
	α_*h*_(*V*) = 0.6 exp(−(*V* + 57)/18.0)
	β_*h*_(*V*) = 0.6 exp((*V* + 57)/13.5)
Membrane capacitances	*C*_*S*_ = 24 pF, *C*_*N*_ = 0.2 pF
Leak conductances	*g*_*L*_^*S*^ = 48 nS, *g*_*L*_^*N*^ = 2 nS
K_LVA_ conductances	*ḡ*_KLVA_^*S*^ = 192 nS, *ḡ*_KLVA_^*N*^ = 8 nS
K_HVA_ conductance of the node	*ḡ*_KLVA_^*N*^ = 450 nS
Na conductance of the node	*ḡ*_Na_^*N*^ = 1500 nS
Reversal potential of leak current	*E*_*L*_ = −60 mV
Reversal potential of potassium current	*E*_*K*_ = −75 mV
Reversal potential of Na current	*E*_Na_ = +35 mV
Reversal potential of synaptic current	*E*_syn_ = 0 mV
Axonal conductance	*g*_axon_ = 118 nS

The kinetics of K_LVA_ and K_HVA_ conductance are taken from the study of chick NM (Rathouz and Trussell, 1998). The kinetics of the Na conductance are based on the data of chick NM (Koyano et al., 1996). All the parameters and equations are the same as we used in our previous study (Funabiki et al., 2011).

In constructing synaptic inputs, half the NM fibers were assumed to be from the ipsilateral side, and the remaining half of the NM fibers from the contralateral side. NM fibers from each side were assumed to be phase-locked to generate the oscillatory conductance, and the NM inputs from the two sides were summed with a phase difference δ (see Figure 7A). The spike rate of the model neuron with δ = 0 was called the “in-phase rate,” while that with δ = π was referred to as the “out-of-phase rate” (see Figure 8A).

### Analytical expressions

Analytical results obtained in the accompanying paper (Ashida et al., 2013) are summarized in Table 1. Equations 1–5 describe the parameter dependence of the average (*D*_*G*_), the signal part (*A*_*G*_), and the noise part (*N*_*G*_) of the synaptic conductance input, as well as the signal (*A*_*V*_) and noise (*N*_*V*_) components of the membrane potential.

### Conflict of interest statement

The authors declare that the research was conducted in the absence of any commercial or financial relationships that could be construed as a potential conflict of interest.

## Abbreviations

ITD: interaural time difference
NM: nucleus magnocellularis
NL: nucleus laminaris
VS: vector strength
EPSG: excitatory post synaptic conductance
K_HVA_: high voltage activated potassium
K_LVA_: low voltage activated potassium
LLDa: anterior part of the dorsal lateral lemniscus
MSO: medial superior olive.
